# A multicenter, prospective trial to evaluate mesh-augmented sacrospinous hysteropexy for uterovaginal prolapse

**DOI:** 10.1007/s00192-014-2564-x

**Published:** 2014-11-14

**Authors:** K. Jirschele, M. Seitz, Y. Zhou, P. Rosenblatt, P. Culligan, P. Sand

**Affiliations:** 1 Division of Urogynecology, University of Chicago/NorthShore University HealthSystem, 9650 Gross Point Road, Suite 3900, Skokie, IL 60076 USA; 2 Center for Biomedical and Research Informatics, NorthShore University HealthSystem, Evanston, IL USA; 3 Boston Urogynecology Associates, Cambridge, MA USA; 4 Atlantic Health System Division of Urogynecology, Morristown, NJ USA

**Keywords:** Hysteropexy, Pelvic organ prolapse, Polypropylene mesh, Surgery

## Abstract

**Introduction and hypothesis:**

Hysterectomy is often part of pelvic organ prolapse repair. However, this may offer no benefit when compared to uterine preservation. We aimed to prospectively evaluate a minimally invasive bilateral sacrospinous hysteropexy using polypropylene mesh. We hypothesized that anatomic success and patient satisfaction can be achieved with this technique.

**Methods:**

Women with uterovaginal prolapse desiring surgery who had completed childbearing were enrolled. Preoperative assessment included standardized prolapse examination and validated symptom and pain scale questionnaires. Women with prior pelvic organ prolapse repair or any contraindication to uterine preservation were excluded. Data including demographic, operative and postoperative information was collected on patients for 1 year following surgery. Continuous variables are summarized as means (standard deviation) and categorical variables are summarized as frequencies and percentages. A mixed-effects model was used to evaluate the changes in questionnaire scores and outcomes at 6 months and 12 months after surgery with random effects accounting for the center effect with adjustment for age.

**Results:**

The study group comprised 99 women from three female pelvic medicine and reconstructive surgery (urogynecology) centers. The average age of the participants was 67.0 years (11.32 years), BMI 26.04 kg/m^2^ (3.56 kg/m^2^), and the majority were multiparous (98.9 %) and menopausal (90.9 %). Overall success at 12 months, as measured by composite outcome was 97.7 % (with the Ba point as the anatomic landmark) and 96.6 % (with the C point as the anatomic landmark). The overall exposure rate was 6.52 % and reoperation rate was 7.53 %. All subjective questionnaire scores and anatomic outcomes had improved at 12 months.

**Conclusions:**

Sacrospinous hysteropexy using a minimally invasive polypropylene mesh kit is an effective and safe technique for addressing uterovaginal prolapse as an alternative to hysterectomy at the time of pelvic reconstructive surgery.

## Introduction

Minimally invasive surgical techniques are being applied to an ever widening array of conditions that previously required major surgical intervention. Alternatives to hysterectomy exist for many bothersome pelvic complaints such as endometrial ablation for menorrhagia and vascular embolization for fibroids. Uterovaginal prolapse is the primary indication for the majority of vaginal hysterectomies performed in the US. Of the more than 117,000 benign vaginal hysterectomies, 44 % were done for prolapse [1]. Although hysterectomy has often been performed reflexively during prolapse surgery there is a growing belief that this strategy may, for some women, offer no specific benefit when compared to newer minimally invasive alternatives.

The aims of this multicenter study were to evaluate anatomic success, patient satisfaction, condition-specific quality of life, morbidity, and recuperation after minimally invasive sacrospinous hysteropexy in women presenting with uterine prolapseof stage II or more. The primary aim was to evaluate the success of the sacrospinous hysteropexy procedure using a composite measure of subjective and objective outcomes. The secondary aims were to determine patient satisfaction, quality of life, morbidity, and recuperation. The procedure was performed in women electing to undergo surgical repair of their prolapse using a uterus-sparing approach, and was evaluated using standardized measures and validated questionnaires in three major female pelvic medicine and reconstructive surgery (urogynecology) referral centers. We wanted to evaluate whether this technique should be considered an initial approach in the surgical treatment algorithm for uterine prolapse.

## Materials and methods

After IRB approval at all institutions, subjects were recruited from three established referral centers for urogynecology and reconstructive pelvic surgery: (1) NorthShore University HealthSystem, Evanston, IL, (2) Atlantic Health System Division of Urogynecology, Morristown, NJ, and (3) Boston Urogynecology Associates, Cambridge, MA. Logistical operations and data analysis were managed at the NorthShore site.

The sample comprised 100 women with uterine prolapse electing to undergo reconstructive vaginal surgery for that condition, who selected uterus-sparing prolapse repair after being familiarized with the potential benefits and risks of the procedure and signing appropriate consents. Multichannel urodynamic testing was performed within 6 months prior to study enrollment in all patients with pelvic prolapse of stage II or more and/or urinary incontinence symptoms. Women were excluded if they had a history of previous vaginal, abdominal or laparoscopic repair for pelvic organ prolapse; cervical dysplasia, gynecologic cancer, undiagnosed irregular vaginal bleeding, endometriosis, or chronic pelvic pain; language barrier that precluded consent or questionnaire completion; or plans for future childbearing. Information was collected on all study patients for 1 year after their operation. The primary outcome was a composite of objective (POP-Q points Ba less than −1 and C less than −½ TVL) and subjective measures (negative response to “Do you experience the feeling of bulging or protrusion in the vaginal area?”).

Women were enrolled and preoperative data were collected including demographic information, POP-Q measurements, and baseline subjective questionnaire results. Surgery was performed at each center by fellowship trained female pelvic medicine and reconstructive surgeons. All enrolled patients underwent hysteropexy, and anti-incontinence and other concomitant procedures at the surgeon’s discretion. Postoperative data were collected. FACES pain scale measurements were collected in the postanesthesia care unit and 24 hours after surgery. Patients were subsequently followed at 2, 6, 12, 26, and 52 weeks after surgery. A nonblinded pelvic examination was performed by surgeon or fellow including POP-Q, and validated subjective measures (Pelvic Floor Distress Inventory, PFDI, and Pelvic Organ Prolapse/Urinary Incontinence Sexual Questionnaire, PISQ) were collected at 6 and 12 months.

The study evaluated mesh-augmented sacrospinous hysteropexy. Each sacrospinous ligament is approached through a standard anterior vaginal incision, and the suspension procedure is performed with no entry into the peritoneal cavity. This anterior sacrospinous technique, and its anatomic benefits, have been previously evaluated [2]. The suspension is bilateral, thus maintaining vaginal length and width without deviation or narrowing of the vagina as might occur with a traditional (unilateral) sacrospinous repair. This hysteropexy technique utilizes a commercially available, soft, polypropylene mesh (Uphold®; Boston Scientific Corporation). Polypropylene mesh repairs allow surgeons to compensate for weak tissues by enhancing scar formation and providing a permanent barrier [3]. This “minimal mesh” technique interposes a relatively small segment of soft polypropylene mesh to reinforce the vaginal apex and adjacent cervix to the sacrospinous ligament bilaterally, addressing the most susceptible areas for possible recurrence. The technique is specifically designed to eliminate placement of any excess mesh in anatomic areas where it would offer no benefit. The procedure is performed through a small horizontal incision in the distal anterior vaginal wall to eliminate overlapping of the mesh with the vaginal suture line to minimize mesh extrusion.

Continuous variables are summarized as means (standard deviation) or median (range) where considered appropriate, and categorical variables are summarized as frequencies and percentages. The summary statistics are presented for the whole sample and then for each center separately. Patient demographics, preoperative physical examination findings, preoperative subjective scores, and perioperative and surgical data are compared across centers (Table 1). One-way ANOVA or the Kruskal-Wallis test was used to compare continuous variables and the Chi-squared test was used to compare categorical variables. A mixed-effects model was used to compare changes from before surgery at 6 months and at 12 months with time as fixed effect and center as random-effect taking into account the hierarchical structure (patients nested in centers) with age and menopause status as covariates where appropriate. The Tukey-Kramer method was used to adjust *p* values for the multiple comparisons. Statistical analysis was performed using SAS 9.3 for Windows (Cary, NC, USA) with *p* < 0.05 considered as statistically significant.

**Table 1:** Preoperative physical examination findings, preoperative subjective questionnaire scores, and perioperative and surgical data

	All centers (*n* = 99)		Atlantic Health System (*n* = 39)		NorthShore University HealthSystem (*n* = 55)		Boston (*n* = 5)		*p* value
	Mean ± SD	Median (range)	Mean ± SD	Median (range)	Mean ± SD	Median (range)	Mean ± SD	Median (range)	
POP-Q points									
Aa	1.32 ± 1.62	2 (−3 – 3)	1.85 ± 1.46	2 (−3 – 3)	1.1 ± 1.62	1 (−3 – 3)	−0.4 ± 1.08	0 (−2 – 0.5)	0.0022*
Ba	2.05 ± 2.29	2 (−3 – 9)	2.79 ± 2.2	3 (−2 – 9)	1.71 ± 2.27	2 (−3 – 7.5)	0 ± 0.61	0 (−1 – 0.5)	0.0042*
C	−0.94 ± 3.62	−1 (−9 – 9)	0.03 ± 3.58	0 (−6 – 9)	−1.65 ± 3.57	−3 (−9 – 7.5)	0.8 ± 3.49	−1 (−5 – 4)	0.0603
Gh	3.94 ± 1.35	4 (1 – 7)	3.38 ± 1.61	3 (1 – 7)	4.34 ± 1	4.5 (2.5 – 6.5)	3.9 ± 1.24	4 (2 – 5)	0.0029*
pb	2.85 ± 1.03	3 (1 – 6)	2.17 ± 0.83	2 (1 – 5)	3.26 ± 0.84	3 (1 – 5.5)	3.6 ± 1.56	3.5 (2 – 6)	<0.0001*
tvl	9.4 ± 1.38	9.5 (2.5 – 13)	9.49 ± 0.91	9.5 (8 – 12)	9.49 ± 1.4	9.5 (3 – 13)	7.7 ± 2.99	9 (2.5 – 10)	0.2228
Ap	−1.02 ± 1.52	−1 (−5 – 9)	−0.67 ± 1.15	−1 (−2 – 3)	−1.11 ± 1.81	−1.5 (−3 – 9)	−2.8 ± 1.3	−2 (−5 – 2)	0.0009*
Bp	−0.68 ± 1.98	−1 (−3 – 8)	−0.54 ± 1.79	−1 (−2 – 8)	−0.62 ± 2014	−1 (−3 – 6)	−2.4 ± 0.55	−2 (−3 – 2)	0.0079*
D	−3.48 ± 3.32	−4 (−9.5 – 9)	−1.09 ± 3.32	−1 (−7 – 9)	−5.13 ± 2.23	−5 (−9.5 – 5)	−4 ± 2.32	−2.5 (−7 – 2)	<0.0001*
PISQ-12 total score^a^	30.99 ± 6.93	30 (18 – 48)	31.65 ± 4.98	33 (22 – 39)	31.3 ± 7.54	30 (18 – 48)	25.67 ± 1.15	25 (25 – 27)	0.3250
PFDI summary score	66.52 ± 47.51		60.91 ± 39.61		67.71 ± 51.05		99.17 ± 65.16		0.4160
POPDI-6	33.52 ± 20.53		32.91 ± 20.14		33.42 ± 20.98		39.17 ± 22.94		0.7942
CRADI-8	16.5 ± 17.11		14 ± 14.68		17.14 ± 17.97		30 ± 23.03		0.3116
UDI-6	33.67 ± 24.45		28.1 ± 23.37		36.37 ± 24.69		51.83 ± 21.23		0.0617
Estimated blood loss (cm^3^)	112.22 ± 94.74		78.21 ± 56.25		129.55 ± 97.79		187.00 ± 195.82		0.0058*
Extrusion rate, *n* (%)	6 (6.52)		3 (8.82)		3 (5.66)		0 (0.00)		
Reoperation rate, *n* (%)	7 (7.53)		3 (8.82)		4 (7.41)		0 (0.00)		

*PISQ* Pelvic Organ Prolapse/Urinary Incontinence Sexual Questionnaire, *PFDI* Pelvic Floor Distress Inventory, *POPDI* Pelvic Organ Prolapse Distress Inventory, *CRADI* Colorectal-Anal Distress Inventory, *UDI* Urinary Distress Inventory

*p < 0.05

^a^PISQ-12 has a range from 0 to 48 with higher scores indicating better sexual function

## Results

A total of 99 women were enrolled in the study across three centers and underwent sacrospinous mesh-augmented hysteropexy for uterovaginal prolapse. Demographic data are presented in Table 2. The average age of the participants was 67.0 ± 11.32 years, BMI was 26.04 ± 3.56 kg/m^2^, and the majority were multiparous 98 (98.9 %) and menopausal 90 (90.9 %). All bilateral hysteropexy procedures were performed through an anterior approach. In addition 83 % of the enrolled participants had concomitant native tissue posterior repair and 85 % had midurethral sling. Complete subjective and objective data was available for 87 % of the enrolled participants at 12 months. The overall success rate at 12 months as measured by the composite outcome was 97.7 % (with the Ba point as the anatomic landmark) and 96.6 % (with the C point as the anatomic landmark). In addition, calculating outcome to include any anterior, apical, or posterior compartment POP-Q point <0 and negative response to the PFDI question on bulge, the success rate was 97.7 %.

**Table 2:** Patient characteristics of the study sample combining data from all centers

	All centers (*n* = 99)	*p* value
Age (years)	67.02 ± 11.32	0.0144*
BMI (kg/m^2^)	26.04 ± 4.35	0.3629
Menopause (%)	90	0.0430*
Sexually active (%)	33	0.2160
Parity	2.92 ± 1.45	0.5028

*p < 0.05

All POP-Q measurements had improved at 12 months. The mesh extrusion rate was 6.52 % across all sites. Of the patients with polypropylene mesh exposure, many were asymptomatic (three of seven) and required no treatment. One was managed with successful office excision, one required surgery for excision, and two were managed with vaginal estrogen therapy. The reoperation rate was 7.53 %. Two of six had recurrent prolapse in the posterior compartment requiring posterior colporrhaphy. Two patients had sling revisions. One patient had the mesh excised and another had a vaginal hysterectomy for recurrent apical prolapse.

All PFDI subjective questionnaire scores and anatomic outcomes improved at both 6 months and 12 months (Table 3). The PFDI scores at 12 months compared with those before surgery were as follows: summary score −40.3 ± 4.5 (*p* < .0.0001), Pelvic Organ Prolapse Distress Inventory-6 −27.1 ± 2.1 (*p* < 0.0001), Colorectal-Anal Distress Inventory-8 −6.6 ± 1.7 (*p* = 0.0003), and Urinary Distress Inventory-6 −25.1 ± 2.5 (*p* < .0.0001). The PISQ data were not robust enough to analyze (<25 % of patients had complete PISQ data at 12-months).

**Table 3:** POP-Q measurements and PFDI scores from before surgery to 6 months and 12 months after surgery

	6 months			12 months		
	Mean ± SE	95 % confidence Interval	*p* value	Mean ± SE	95 % confidence Interval	*p* value
POP-Q points						
Aa	−3.53 ± 0.16	(−3.91, −3.14)	<0.0001*	−3.49 ± 0.15	(−3.86, −3.11)	<0.0001*
Ba	−4.24 ± 0.20	(−4.73, 3.75)	<0.0001*	−4.19 ± 0.20	(−4.67, −3.72 )	<0.0001*
C	−5.94 ± 0.38	(−6.85, −5.03)	<0.0001*	−5.90 ± 0.37	(−6.80, −5.01)	<0.0001*
Gh	−1.25 ± 0.13	(−1.58, −0.92)	<0.0001*	−1.23 ± 0.13	(−1.55, −0.90)	<0.0001*
pb	0.56 ± 0.09	(0.33, 0.78)	<0.0001*	0.51 ± 0.08	(0.30, 0.72)	<0.0001*
tvl	0.07 ± 0.14	(−0.26, 0.40)	0.8611	0.06 ± 0.13	(−0.27, 0.38)	0.9102
Ap	−1.62 ± 0.16	(−2.01, 1.22)	<0.0001*	−1.51 ± 0.16	(−1.89, −1.12)	<0.0001*
Bp	−1.81 ± 0.19	(−2.28, −1.35)	<0.0001*	−1.79 ± 0.19	(−2.25, −1.34)	<0.0001*
D	−4.84 ± 0.37	(−5.72, −3.97)	<0.0001*	−4.70 ± 0.36	(−5.57, −3.82)	<0.0001*
PFDI summary score	−43.47 ± 4.59	(−54.35, −32.58)	<0.0001*	−40.27 ± 4.54	(−51.03, −29.51)	<0.0001*
POPDI-6	−28.00 ± 2.07	(−32.93, −23.08)	<0.0001*	−27.08 ± 2.05	(−31.94, −22.22)	<0.0001*
CRADI-8	−7.69 ± 1.67	(−11.64, −3.73)	<0.0001*	−6.55 ± 1.65	(−10.47, −2.64)	0.0003*
UDI-6	−24.28 ± 2.39	(−29.95, −18.62)	<0.0001*	−25.12 ± 2.47	(−30.74, −19.50)	<0.0001*

*PFDI* Pelvic Floor Distress Inventory, *POPDI* Pelvic Organ Prolapse Distress Inventory, *CRADI* Colorectal-Anal Distress Inventory, *UDI* Urinary Distress Inventory

*p < 0.05

## Discussion

As specialists in recent years have begun to more strongly focus on uterus-preserving techniques, it has become more widely acknowledged that removing the uterus may not be necessary or beneficial for prolapse without intrauterine pathology. It has been proposed that uterus-sparing prolapse repair may provide several potential advantages including avoidance of vaginal shortening following hysterectomy with possible sexual dysfunction, reduced dissection and nerve trauma, and reduced disruption of connective tissues along the vaginal apex including the uterosacral cardinal ligament complex and paracervical ring. These structures are thought to contribute to maintenance of normal pelvic support, and leaving them intact may help prevent recurrent vaginal vault prolapse and/or enterocele.

From the patient perspective, across the age spectrum, uterus-sparing surgery is an attractive alternative to a traditional hysterectomy-based repair. Many women with uterovaginal prolapse avoid or delay an operation because they wish to avoid hysterectomy because of its potentially negative impact on their quality of life. In some women the desire to avoid hysterectomy relates to a reluctance to give up an organ so closely associated with their femininity and reproductive health. Others may have uncertain plans for possible future childbearing, concern about the invasiveness of the procedure, recuperation, or fear of diminished sexual function. Uterus-sparing prolapse techniques therefore have the potential to provide both physical and psychological benefits to many women.

Prior observational studies have suggested that sacrospinous hysteropexy is a safe and effective alternative to hysterectomy, with low morbidity and good patient satisfaction. These studies have generally indicated more rapid recovery than following hysterectomy, with less surgical morbidity and blood loss, shorter operating time, significantly less postoperative pain, and shorter hospital stay [4–7]. Kovac and Cruikshank [4] found that unilateral sacrospinous hysteropexy was associated with substantially less intraoperative morbidity than hysterectomy. Hefni et al. [5] compared uterine preservation with hysterectomy and found equally high rates of success (95 % vs. 96 %). Maher et al. [6] compared sacrospinous hysteropexy with vaginal hysterectomy. At a mean follow-up of 33 months, the rate of apical recurrence after hysteropexy (3.6 %) was no higher than after vaginal hysterectomy. The uterus-sparing technique was associated with lower mean estimated blood loss (198 vs. 402 cm^3^), shorter operative time (59 vs. 91 min), and equal patient satisfaction (85 % vs. 86 %). Van Brummen et al. [7] compared sacrospinous hysteropexy with vaginal hysterectomy, and found that hysterectomy was associated with a threefold higher rate of urgency urinary incontinence and overactive bladder syndrome. Women undergoing hysteropexy reported a quicker recovery than those undergoing hysterectomy, and there were no differences in anatomic outcomes or prolapse recurrence rates. In a prospective randomized trial, Dietz et al. compared 66 women after either vaginal hysterectomy or sacrospinous hysteropexy and concluded that recovery was earlier after hysteropexy, and despite a higher rate of recurrent apical prolapse there were no differences in functional outcomes or quality of life [8].

Pilot data on 33 graft-augmented hysteropexy procedures indicated high rates of success with no detectable complications after a follow-up of 1 year [9]. More recently, Vu et al. reported data from the use of this procedure that indicated an anterior apical (Aa) recurrence rate of 1.89 %, with no posterior (Ba ≥ −1) or apical (C ≥ 0) recurrences [10]. These results were comparable to those in women who had post-hysterectomy vault prolapse and concomitant vaginal hysterectomy. The overall mesh complication rate in this cohort was 2.6 % [10]. Our findings suggest that sacrospinous hysteropexy using polypropylene mesh is an efficacious option for the surgical management of pelvic organ prolapse. Success rates with a composite outcome after 1 year using this technique were excellent with good anatomic and subjective results. Composite outcomes that seek to measure success from various perspectives have recently gained favor [11]. The mesh exposure rate in this multicenter trial of 6.5 % is in line with previously published rates but higher than previously reported rates with this technique [10], and this result may be more representative of anticipated results throughout the world. Mesh exposure rates after vaginal surgery augmented with mesh are in the range 3 – 16 %.

Mesh exposure rates generally vary and are in the range 3.6 – 18 %. Dwyer and O’Reilly reported a rate of 9 % [12]. Davila and Jijon estimated the rate at 10 % [13] and Khandwala and Jayachandran reported a rate of 3.6 % [14]. Achtari et al. reported a rate of 7 % with polypropylene mesh [15]. Gutman et al. reported a rate of 15.6 % with Prolift® (Ethicon, Inc) [16]. The Cochrane review of surgical management of pelvic organ prolapse in women indicates a rate of 18 % with 9 % of patients undergoing surgical correction for mesh complications [17].

We recognize the controversy surrounding the surgical repair of pelvic organ prolapse using transvaginal polypropylene mesh. We acknowledge that there are specific complications related to the use of vaginal mesh and each enrolled patient was carefully counseled on the benefits and risks associated with a polypropylene mesh repair. These potential complications need to be seriously considered by both physician and patient. Our experience suggests that when surgeons are properly trained and patients are appropriately selected there are minimal related complications.

The study had several strengths, including the prospective, multicenter design. The presentation of the primary outcome as a composite of objective and subjective measures helps define success in anatomic and clinically relevant ways that may be more important to the patient.

The limitations of the study include a design without a comparison arm or specific power calculations to enhance statistical robustness. Another limitation was relatively short-term results, with lack of follow up beyond 12 months. Examinations were not universally completed by a third party and this was a potential source of bias. Our study population was primarily Caucasian, relatively homogeneous, and lacked racial diversity. Although a multicenter study enrollment among sites was not consistent.

In conclusion, sacrospinous hysteropexy using a minimally invasive polypropylene mesh kit was found to be an effective and safe technique for addressing uterine prolapse as an alternative to hysterectomy at the time of pelvic reconstructive surgery when assessed with a composite outcome.
